# Objective measures of physical activity and physical capacities in lipedema - a scoping review

**DOI:** 10.1186/s12905-026-04271-y

**Published:** 2026-01-15

**Authors:** Ida Åström Malm, Paola Violasdotter Nilsson, Anita Hurtig-Wennlöf

**Affiliations:** 1 Department of Clinical Diagnostics, School of Health and Welfare, Jönköping University, Jönköping, Sweden; 2 Department of University Library, Jönköping University, Jönköping, Sweden

**Keywords:** Lipedema, Objective physical activity assessment, Functional capacity

## Abstract

**Background:**

Lipedema is a chronic condition characterized by abnormal subcutaneous fat accumulation, predominantly affecting women. It is associated with pain, swelling, reduced mobility, and psychological distress, all of which may limit physical activity (PA) and impair physical function. While PA is increasingly recognized as a key component in conservative management, most existing studies rely on self-reported data, which are prone to bias. Objective assessments of PA and physical capacity are essential to better understand functional limitations and guide tailored interventions.

**Objective:**

This scoping review aimed to map and summarize existing studies that have used objective methods to assess physical activity levels and physical function in women with lipedema.

**Methods:**

The review is reported following the PRISMA-ScR guidelines. A systematic search was conducted in five databases (Scopus, Web of Science, MEDLINE, CINAHL, and AMED) from inception to August 13, 2025. Studies were included if they investigated lipedema and used objective measures of PA or physical capacity. After screening 5,147 records and assessing 46 full-text articles, six studies met the inclusion criteria.

**Results:**

The included studies employed various objective tools, such as accelerometry, six-minute walk tests, sit-to-stand tests, and handheld dynamometry. Sample sizes ranged from 31 to 96 participants with lipedema, with a predominance of female subjects. Findings indicated that individuals with lipedema generally exhibit lower walking capacity and muscle strength compared to healthy or BMI-matched controls. Structured exercise interventions, particularly multimodal programs combining aerobic and resistance training, were associated with improvements in walking distance, muscle strength, and pain reduction. However, methodological heterogeneity and small sample sizes limit the generalizability of findings.

**Conclusions:**

Objective assessments reveal that individuals with lipedema have reduced physical capacity, but structured physical activity interventions may yield functional benefits. Despite promising results, the evidence base remains limited. Future research should prioritize standardized, objective measurement protocols and larger, well-designed trials to inform evidence-based guidelines for physical activity in this population.

**Supplementary Information:**

The online version contains supplementary material available at 10.1186/s12905-026-04271-y.

## Introduction

Lipedema is a chronic condition characterized by abnormal subcutaneous fat accumulation, predominantly affecting women. This disorder substantially impairs physical capacity through a variety of interrelated symptoms. A primary feature of lipedema is persistent pain and tenderness, especially upon palpation, which makes physical movement and exercise uncomfortable and often intolerable [1, 2]. Many individuals also experience a sensation of heaviness and tightness in the affected limbs, which further compromises mobility and limits engagement in physical activity (PA) [3, 4]. In addition, lipedema is associated with symmetrical swelling and disproportionate fat distribution, particularly in the lower extremities. This abnormal fat accumulation contributes to physical disfigurement and functional impairment [2, 5].

The pathological fat deposition, particularly around the thighs and knees, often leads to mobility limitations, reduced walking ability, and decreased functional capacity. Chronic fatigue is also prevalent among patients, often intensified by the physical and emotional toll of the disease [2, 6]. This fatigue significantly decreases energy levels and the ability to participate in physical activities [6]. In addition to the physical burden, lipedema frequently has a psychological impact. Symptoms such as anxiety and depression are common and can further reduce motivation and capacity for physical engagement [3, 4, 6, 7]. Collectively, these symptoms may result in limitations to physical functioning and quality of life for those affected by lipedema.

PA is increasingly recognized as an essential component in the conservative management of lipedema. According to the guidelines for the management of lipedema [8], low-impact physical activities such as walking, swimming, and cycling are recommended to improve lymphatic flow, and reduce discomfort. These recommendations are consistent with international guidelines from the World Health Organization (WHO) [9] which emphasize the importance of regular moderate-intensity PA for cardiovascular health, functional capacity, and psychological well-being.

PA is often included in weight management strategies; however, in lipedema, conventional weight loss interventions have little or no effect on the pathological adipose tissue [8]. Consequently, the primary rationale for recommending PA in individuals with lipedema is not weight reduction, but rather improvements in physical function, pain management, and overall health, all of which could support the resilience to live with lipedema. In this context, strictly controlled PA intervention studies may also contribute to a better understanding of functional and physiological responses associated with lipedema [10].

A recent consensus report by Annunziata et al. (2024), published on behalf of the Italian Society of Sports Medicine (SISMe), emphasizes that structured physical exercise should be considered a central therapeutic component in lipedema management rather than a supplementary intervention [11]. The report highlights the benefits of individualized, multimodal exercise programs combining aerobic activity, resistance training, and mobility exercises to improve lymphatic flow, muscle strength, and overall functional capacity. Furthermore, SISMe recommends integrating exercise with complementary therapies—such as compression treatment and nutritional counseling—to optimize outcomes and enhance patients’ quality of life. This aligns with recent evidence showing that exercise-based interventions produce improvements in both objective measures like walking distance and muscle strength, as well as patient-reported outcomes such as pain reduction and improved well-being [11].

Despite the recognized benefits of PA, much of the existing literature on PA and functional limitations in lipedema is based on self-reported outcomes, such as questionnaires assessing mobility loss, pain, and perceived physical functioning [6, 7, 12]. Commonly used tools such as activity questionnaires and symptom diaries are prone to both under- and overestimation actual PA behavior, limiting the accuracy of previous findings in lipedema research [13]. Objective tools such as accelerometry provide standardized and reproducible measures of habitual PA behavior. Performance-based tests, such as the six-minute walk test (6MWT), assess functional physical capacity and allow estimation of aerobic fitness, making these methods sensitive to changes in PA patterns and physical function over time [14]. These methods are particularly relevant in lipedema, where pain, fatigue, and mobility limitations may influence both perceived and actual PA in complex ways. However, to date it remains unclear how often objective measurement methods have been used in lipedema research, what types of tools have been applied, and which outcomes the objective methods have generated. This represents an important gap in the literature.

Lipedema predominantly affects women, with an estimated prevalence almost exclusively in the female population. Given the strong female predominance and the substantial impact of lipedema on physical function, quality of life, and participation in daily activities, a focused synthesis of objectively measured PA and physical capacity is highly relevant within a women’s health perspective.

Therefore, the aim of this scoping review is to map and summarize existing studies that have used objective methods to assess PA levels and physical function in women with lipedema. Particular attention is given to the types of measurement tools used, reported outcomes, and comparison to other populations included in the same studies when applicable.

## Methods

### Design

The scoping review methodology was chosen for allowing a comprehensive overview of the existing literature by employing a flexible and structured approach, suitable for exploring emerging and conceptually broad research areas. The process includes several key phases: defining the research questions, systematically identifying and selecting relevant studies, organizing and charting the data, and finally synthesizing and presenting the findings in a descriptive format [15]. The result is reported following the PRISMA ScR guidelines [16].

### Eligibility criteria

Studies investigating lipedema and objectively measured physical PA were included. Objective measures of physical function were not required for inclusion but were extracted and reported when available in the PA studies. Review articles, opinion papers and letters, were excluded as were non-English language papers.

### Search strategy

**Fig. 1:**
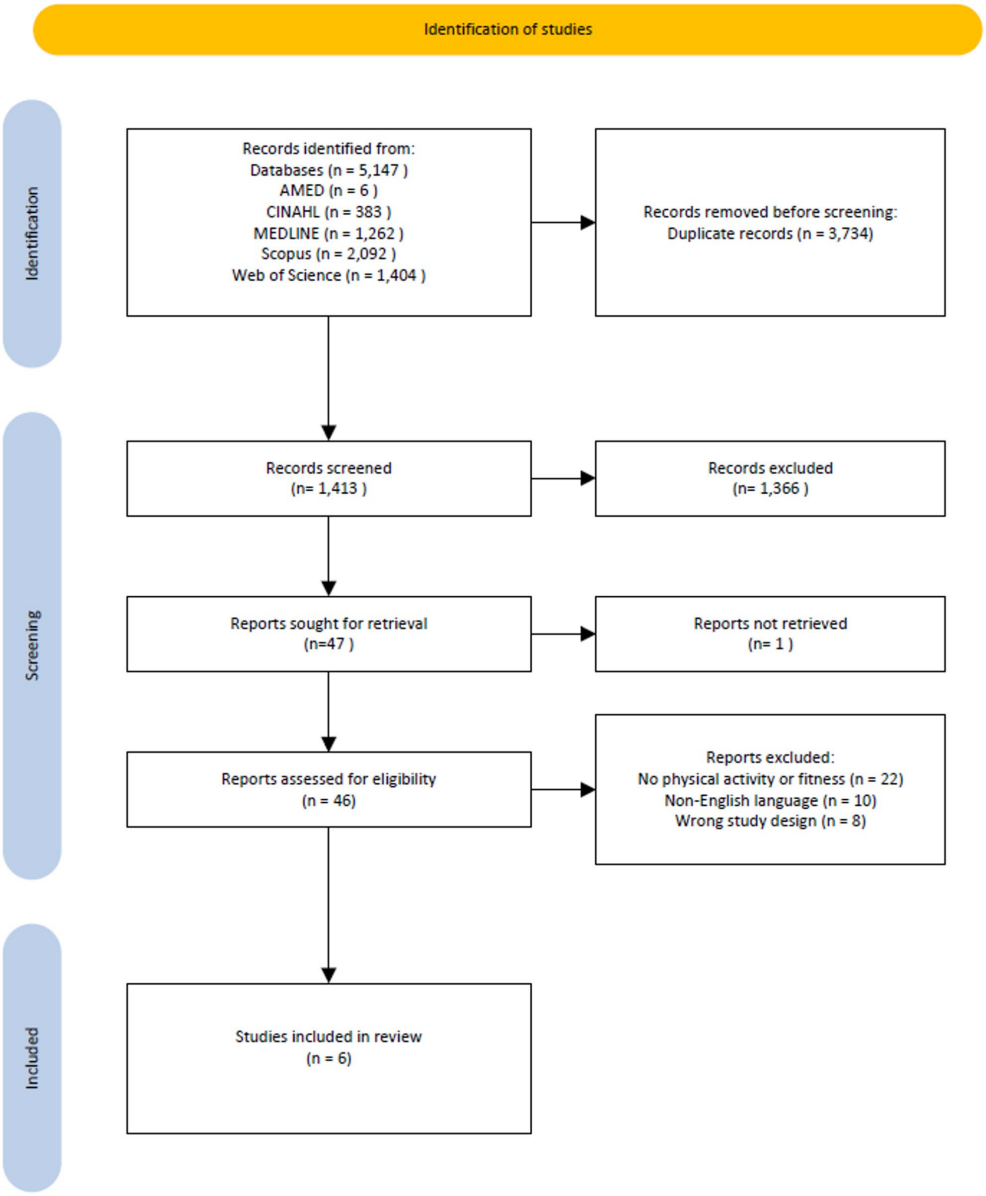
Flowchart of the article selection process

To achieve full comprehensiveness, acknowledging the limited number of published studies on lipedema, the research team decided to manually examine each study addressing lipedema to discover any relation to PA in any form. The search strategy was developed based on controlled terms for lipedema in databases where controlled terms were available, combined with uncontrolled terms covering different spelling variations (lipedema and lipoedema). Five bibliographic databases were searched: AMED (EbscoHost), CINAHL with FullText (EbscoHost), MEDLINE (Ovid), Scopus (Elsevier), and Web of Science Core Collection (Clarivate). No limits regarding publication type, or date were applied. The initial search was conducted on March 5, 2025, and updated on August 13, 2025. After removing duplicates, 1,413 records remained for screening. Of these, 1366 records were excluded based on relevance to the research question. The remaining 46 studies were retrieved in full text and assessed for eligibility. After full-text review, 40 studies were excluded for not meeting the inclusion criteria. Finally, six articles were identified and their methodological level was described using the Pyramid of Evidence principle as outlined by Forsberg and Wengström [17]. The flowchart of included studies is presented in Fig. 1. The full search strategy for all databases is provided in Supplementary file 1.

### Study selection

Two reviewers independently screened all titles, abstracts, and full-text articles. Any discrepancies in study inclusion were discussed and resolved through consensus.

## Results

### Study selection and characteristics of included studies

Six studies met the inclusion criteria for this review. A detailed overview of study characteristics, objective assessment methods, and key outcome measures is presented in Table 1, which serves as the primary source for numerical comparisons across studies. The included studies represent a variety of methodological designs, including cross-sectional studies [18, 19], randomized controlled trial [20, 21], longitudinal observational study [22], and open observational cohort design [23]. Only one of the articles reporting power calculations [21]. However, the transferability was categorized as low due to a very specific intervention method (decongestive therapy), resulting in moderate quality. Remaining included articles were assessed as low quality due to study design, no power calculation, and mostly few observations [17], see Table 1,

**Table 1:** Summary of studies using objective methods to assess physical activity and physical function in lipedema

Authors (Year, Country)	Title	Study Aim	Design/Sample Size (*n*)	Mean Age (SD)/Gender	Objective PA Tool	Main Results	Interpretation (PA)	Critical assessment
Angst F, Lehmann S, Aeschlimann A, Sandòr PS, Wagner S. Switzerland, 2020 [18]	Cross-sectional validity and specificity of comprehensive measurement in lymphedema and lipedema of the lower extremity: a comparison of five outcome instruments	To examine the validity of outcome measures and their ability to distinguish between lymphedema and lipedema.	Cross-sectional design Lipedema *n* = 96 Lymphedema *n* = 107	Lipedema Age: 46,7 (± 13,7) years female 100%, Lymphedema Age: 56,4 (± 14,8) years female 71%	Six-minute Walk test	Participants with lymphedema walked a longer average distance (463.7 m) compared to those with lipedema (426.1 m). However, the difference was not statistically significant (adjusted SMD = 0.28; 95% CI: −0.05 to 0.60; *p* = 0.096).	Although participants with lymphedema walked farther on average than those with lipedema, the lack of statistical significance suggests that this difference may not reflect a true difference in physical capacity.	Low
Atan T, Bahar-Özdemir Y Turkey, 2021 [21]	The Effects of Complete Decongestive Therapy or Intermittent Pneumatic Compression Therapy or Exercise Only in the Treatment of Severe Lipedema: A Randomized Controlled Trial	Compare the effects of exercises combined with decongestive therapy or intermittent pneumatic compression therapy or alone in patients with severe lipedema.	Single-center, prospective, single-blinded, randomized controlled trial. *n* = 31	Age 59,10 (± 7,37) years. Female 100%	Six-minute Walk test	Baseline means 6-minute walk distance was 317.3 m (SD = 125.8). All groups showed improvements in walking capacity over time (*p* < 0.001), with the most substantial gains observed in the CDT plus exercise and IPCT plus exercise groups.	Exercise combined with CDT or IPCT appears to enhance walking capacity in individuals with severe lipedema, suggesting that multimodal interventions may be effective for improving physical function.	Moderate
Benz T, Lehmann S, Sandor PS, Angst F. Switzerland, 2023 [22]	Relationship between subjectively-rated and objectively-tested physical function across six different medical diagnoses.	To examine the relationship between self-reported and performance-based physical function (SF-36 and 6MWT) across diagnoses, including the influence of pain and mental health.	Multicenter longitudinal observational study. *n* = 445 Lipedema *n* = 27 Whiplash *n* = 71 Low back pain *n* = 121 Fibromyalgia *n* = 84 Lymphoedema *n* = 78 Post-acute coronary syndrome *n* = 64	Lipedema 50.8 (± 13,8) years Female 100% Whiplash 40.7 (± 12.2) years Female 64,8% Low back 49.0 (± 12.1) years Female 61,2% Fibromyalgia 48.5 (± 9.4) years Female 88,1% Lymphedema 59.4 (± 15.3) years Female 78,2% Post-acute coronary syndrome 65.9 (± 10.2) years Female 21,9%	Six-minute Walk test	Individuals with lipedema showed an average increase of 48.5 m in 6-minute walk distance (6MWD) from baseline (414.3 m) to follow-up (469.1 m). While they walked shorter distances than patients with lymphedema at both times, the magnitude of improvement was comparable. A stronger correlation between self-reported physical function and 6MWD was observed in the lipedema group (*r* = 0.791), suggesting good agreement between perceived and actual physical capacity.	Despite lower absolute walking distances, individuals with lipedema demonstrated comparable functional improvements to those with lymphedema. The strong correlation with self-reported function suggests good alignment between perceived and actual physical capacity	Low
José van Esch-Smeenge, Robert J Damstra, Ad A Hendrickx Netherlands, 2017 [19]	Muscle strength and functional exercise capacity in patients with lipoedema and obesity: a comparative study	To compare muscle strength and exercise capacity in lipedema and obesity to identify objective diagnostic criteria.	Cross-sectional pilot study *N* = 44 Lipedema *n* = 22 Obesity *n* = 22	Lipedema 39,23 (± 13,0) years. Obesity 48,45 (± 9,9) years female 100%	Six-minute Walk test Muscle strength of the quadriceps	Women with lipedema had significantly lower quadriceps strength in both legs compared to BMI-matched women with obesity. Although the lipedema group also walked shorter distances in the 6MWT (494.1 m vs. 523.9 m), the difference was not statistically significant.	Reduced muscle strength in lipedema may contribute to lower functional capacity, although walking performance was not significantly different from BMI-matched controls.	Low
Mortimer PS, Pearson M, Gawrysiak P, Riches K, Keeley V, Tew KF, Cranwell EJ. United Kingdom, 2024 [23]	LymphActiv: A Digital Physical Activity Behavior Intervention for the Treatment of Lymphedema and Lipedema	Determine the feasibility of LymphActiv as a treatment option to improve treatment outcomes in lymphedema	Open observational cohort design *N* = 55	54 (± 12,4) years Female 80%	Wrist-worn PA monitor continuously for 7 days.	Wrist-worn activity trackers showed significant increases in physical activity over 24 weeks. Daily moderate-intensity activity rose by 31% (from 39 to 51 min), non-sedentary time increased by 18% (169 to 198 min), and moderate activity bouts (≥ 10 min) showed the largest gain, increasing from 6 to 26 min per day (*p* < 0.05).	Findings suggest that personalized digital support may help promote increased physical activity in individuals with lipedema, particularly in sustained moderate-intensity activity.	Low
Sakizli Erdal E, Ergin C, Haspolat M, Erturk B, Keser I. Turkey,2025 [20]	Effects of multimodal exercise program on edema, pain, exercise capacity, lower extremity muscle strength and function in patients with lipedema	Examine the effects of a multimodal exercise program on edema, pain, exercise capacity, lower extremity muscle strength, and function in patients with lipedema	Randomized controlled trial *N* = 22 Exercise *n* = 11 Control *n* = 11	Exercise 44.45 (± 8.12) years Control 52.27 (± 8.66) years Female: 100%	Six-minute Walk test Sit-to-stand test Handheld dynamometer	Significant improvements in pain, muscle strength, endurance, and functionality in the exercise group. No statistically significant differences between groups.	Structured multimodal exercise (aerobic + strengthening) is beneficial for pain reduction, muscle strength, and functional capacity in lipedema patients.	Low

Sample sizes ranged from 31 to 445 participants, including both genders, although with a predominance of females, reflecting the typical gender distribution of the lipedema population. More specifically, the number of lipedema subjects included ranged from 31 to 96, thus the majority of the subjects included consisted of different comparison groups. The mean age across the studies ranged from 39 to 59 years.

The studies were conducted across four countries: Switzerland (two studies), Turkey (two studies), the Netherlands, and the United Kingdom. Publication years ranged from 2017 to 2025, with a concentration of studies published after 2020 (*n* = 5).

### Findings from the selected publications

The scoping review identified three themes: (1) Objectively Measured PA, Aerobic Capacity and Muscle Strength, (2) Effects of PA and (3) Differences between lipedema and comparison groups within the included studies.

#### Objectively measured physical activity, aerobic capacity and muscle strength

All six included studies reported results based on objective measures of PA and/or muscle strength in individuals with lipedema. A variety of tools and test protocols were used, including wearable activity monitors, walking tests, and muscle strength assessments. Table 1 provides a structured overview of the measures used in each study; the following text synthesizes the main baseline findings across these assessments.

Objectively measured physical activity was reported in one study. In the LymphActiv study [23], daily activity was monitored using a wrist-worn accelerometer. At baseline, participants accumulated an average of 39 min (SD = 38) of moderate-intensity physical activity per day, with 169 min (SD = 82) spent in non-sedentary time. Daily energy expenditure averaged 3883 kcal (SD = 903), and sustained moderate-intensity activity bouts (≥ 10 min) were limited, with a mean duration of 6 min (SD = 13) per day.

Aerobic capacity, assessed using the 6MWT, was reported in five studies. In Angst et al. (2022) [18], individuals with lipedema walked a mean distance of 426.1 m (SD = 114.0). In Benz et al. (2023) [22], baseline 6MWT was 414.3 m (SD = 136.9), and in the study by Atan et al. (2021) [21], the combined baseline 6MWD across participants was 317.3 m (SD = 125.8). van Esch-Smeenge et al. (2017) [19] observed a higher mean baseline distance of 494.1 m (SD = 116.0) in their cross-sectional comparison of women with lipedema and obesity. In the multimodal exercise study by Sakizli Erdal et al. (2025) [20], baseline distances differed between groups, with the exercise group achieving 540 m and the control group 460 m.

Lower-limb muscle strength, measured with handheld dynamometry, was reported in two studies. van Esch-Smeenge et al. (2017) [19] found mean quadriceps strength values of 269.7 N (SD = 67.8) in the right leg and 259.9 N (SD = 77.3) in the left leg. In Sakizli Erdal et al. (2025) [20], baseline quadriceps strength was 20.0 kg in the exercise group and 20.4 kg in the control group. Baseline functional lower-limb performance, assessed using the 30-second sit-to-stand test, showed 11 repetitions in the exercise group and 10 in the control group [20].

Across studies, baseline 6MWT distances ranged from approximately 317 to 540 m, and quadriceps strength values varied depending on assessment method and population characteristics. Objective PA levels were reported in one study and indicated low amounts of sustained moderate-intensity activity.

#### Effects of physical activity in lipedema subjects

Three of the included studies evaluated the effects of structured PA interventions on objectively measured outcomes, including walking capacity, lower-limb muscle strength, pain, and daily activity patterns. This section synthesizes the pre- to post-intervention changes reported in these studies. Complementary baseline values and study characteristics are presented in Table 1.

In the LymphActiv study [23], participants showed marked improvements in accelerometer-measured physical activity over the 24-week intervention period. Moderate-intensity physical activity increased by 31% (from 39 to 51 min per day), non-sedentary time increased from 169 to 198 min per day, and sustained moderate-intensity activity bouts (≥ 10 min) rose from 6 to 26 min per day.

Atan et al. [21] examined the effects of exercise-based rehabilitation combined with complete decongestive therapy (CDT) or intermittent pneumatic compression therapy (IPCT) alone in patients with lipedema. Across all 31 participants, the mean 6MWT distance increased from 318 m at baseline to 371 m post-intervention, corresponding to an overall improvement of approximately 53 m. The greatest gains were observed in the groups receiving multimodal treatment in combination with exercise.

In the six-week multimodal exercise intervention by Sakizli Erdal et al. [20], participants in the exercise group demonstrated improvements across several objectively measured outcomes. Walking distance increased from 540 to 567 m on the 6MWT, the 30-second sit-to-stand test improved from 11 to 13 repetitions, and right quadriceps muscle strength increased from 20.0 kg to 27.8 kg. Reductions in pain during rest and activity were also reported.

Across the included intervention studies, increases in objectively measured PA, walking capacity, and lower-limb muscle strength were consistently reported. Improvements in 6MWT performance ranged from modest increases in exercise-only interventions to larger changes in multimodal programs. Accelerometer measures showed higher daily activity levels and more continuous activity patterns, and dynamometry-based strength assessments demonstrated measurable gains following strengthening components.

#### Differences between lipedema and comparison groups within the included studies

As shown in Table 1, the included studies that directly compare individuals with lipedema to other groups within the same study provide additional insights into how lipedema-related functional limitations differ from those observed in lymphedema or obesity.

Compared to patients with lower limb lymphedema, those with lipedema walked shorter distances on the 6MWT at both baseline (414.3 m vs. 452.2 m) and follow-up (469.1 m vs. 503.5 m). However, both groups demonstrated a similar magnitude of improvement over time (lipedema: +48.5 m; lymphedema: +51.3 m). The correlation between SF-36 Physical Functioning and 6MWD was stronger in the lipedema group (*r* = 0.791) than in the lymphedema group (*r* = 0.642), indicating good alignment between perceived and measured function in lipedema [22].

Angst et al. (2022) reported that participants with lymphedema walked a greater distance (mean = 463.7 m) compared to those with lipedema (mean = 426.1 m) [18]. The unadjusted standardized mean difference (SMD) was 0.30 (95% CI: −0.02 to 0.62, *p* = 0.067), and the adjusted SMD was 0.28 (95% CI: −0.05 to 0.60, *p* = 0.096) [18].

One study compared lipedema subjects with BMI-matched women with obesity [19]. It was found that women with lipedema had significantly lower quadriceps strength compared to the obesity group (left leg: 132.7 ± 40.9 N vs. 167.2 ± 40.2 N, *p* = 0.004; right leg: 135.2 ± 38.4 N vs. 167.4 ± 43.5 N, *p* = 0.012).The lipedema group also walked a shorter distance on the 6MWT (494.1 ± 116.0 m vs. 523.9 ± 62.9 m), although this difference was not statistically significant (*p* = 0.296).

## Discussion

The aim of this scoping review was to map the existing research on PA in individuals with lipedema, with a particular focus on studies that used objective measurement methods. The results indicate that PA has a positive impact on walking capacity, muscle strength, pain, and quality of life in individuals with lipedema. However, several knowledge gaps and methodological limitations were identified, making it difficult to draw clear conclusions.

The included studies used a variety of objective tools such as activity monitors, 6MWT, and dynamometry. These methods provide more reliable data than self-reported measures, which are prone to misclassification of PA intensity, duration, and functional capacity [13].

Although objective assessment methods improve data quality and reduce the risk of misclassification of PA and physical capacity, they are not without limitations. Activity monitors such as accelerometers are subject to methodological challenges related to device selection and placement, epoch length, wear-time compliance, and the application of population-based thresholds that may not adequately capture individual or condition-specific activity related to PA limitations. In addition, variability in wear time and heterogeneity in data processing and analytical approaches can introduce bias and limit comparability across studies [24]. Similarly, even though the 6MWT is widely used to assess functional walking capacity, performance may be influenced by learning effects, use of encouragement, motivation, and disease-related symptoms, as well as variations in test protocols, which can affect reproducibility and the interpretation of functional capacity [25].

Taken together, the included studies suggest that structured PA may be associated with improvements in functional limitations commonly experienced by individuals with lipedema. Improvements in walking capacity, muscle strength, and pain suggest that exercise interventions may help interrupt the cycle of pain, reduced mobility, and physical deconditioning that characterizes the condition. Multimodal approaches that combine aerobic and strengthening components, and that are integrating with other conservative treatments, may be particularly relevant, as they address both functional capacity and symptom burden. From a clinical perspective, the findings based on objectively measured outcomes support the inclusion of tailored PA as a core component of conservative lipedema management, with a primary focus on improving function and daily activity rather than weight loss. Rather than reflecting isolated study outcomes, these patterns suggest a consistent functional response to structured PA across heterogeneous interventions.

The comparative findings reported here stem from two types of pairwise comparisons: lipedema versus lymphedema, and lipedema versus obesity, depending on the study. These comparisons revealed differences in physical capacity between the studied subgroups. Individuals with lipedema generally exhibited shorter walking distances and lower muscle strength compared to BMI-matched control groups, suggesting that lipedema impacts physical function beyond the effects of excess body weight [18, 19, 22]. These comparative findings suggest that the functional limitations observed in lipedema cannot be explained solely by excess body weight or limb swelling. Instead, they point toward condition-specific factors such as pain sensitivity, tissue characteristics, and fatigue that may uniquely constrain physical capacity in lipedema. In this context, objective outcome measures may help clarify which aspects of physical function are most amenable to targeted PA interventions. These findings suggest that structured PA may be relevant for understanding physiological constraints related to pain sensitivity, tissue characteristics, and physical deconditioning.

One important observation is that all the values for 6MWT reported in the lipedema studies were lower than the reference value from a general population of similar age where the expected mean distance is approximately 581 m [26]. None of the studied groups, whether individuals with lipedema or their comparison groups, reached this reference level. Although these findings do not allow causal conclusions, they suggest that reduced walking capacity may be part of the functional profile associated with lipedema. Lower 6MWT performance has been linked to diminished aerobic capacity and reduced ability to sustain daily physical activities [27], which may help explain the mobility challenges frequently reported by individuals with lipedema. Several factors may contribute to these reduced walking distances, including pain-related movement avoidance, early fatigue, increased peripheral load in the lower limbs, and psychosocial factors such as fear of symptom exacerbation. The fact that even comparison groups failed to reach reference values further highlights the complexity of interpreting functional performance in populations with chronic mobility limitations, underscoring the importance of objective outcome measures and standardized test protocols to elucidate physiological capacity alongside perceived exertion.

Several methodological limitations were identified. The included studies were characterized by small sample sizes, considerable heterogeneity in study designs, measurement tools, and intervention protocols, and the lack of long-term follow-up, all of which reduce generalizability and complicate cross-study comparisons. In addition, not all studies incorporated control groups, limiting the ability to distinguish lipedema-specific functional impairments from effects related to comorbidities, obesity, or general deconditioning. Collectively, the overall low methodological quality of the included evidence necessitates cautious interpretation of the findings and restricts conclusions regarding the consistency and magnitude of effects. Finally, although lipedema predominantly affects women, the near absence of male participants limits the evaluation of potential sex-related differences, making it unclear whether these findings can be generalized to men with lipedema.

Future research should prioritize: (1) standardized accelerometry protocols, including device placement, wear-time criteria, and data processing; (2) unified reporting and testing procedures for the 6MWT; (3) standardized dynamometry methods for muscle strength assessment; (4) intervention studies with follow-up periods of at least 6–12 months; and (5) clear documentation of exercise intensity, frequency, duration and progression.

## Conclusion

This scoping review highlights the positive impact of PA on walking capacity, muscle strength, pain reduction, and overall quality of life in individuals with lipedema. Structured exercise interventions, particularly multimodal programs, appear to provide significant benefits for functional capacity and symptom management.

However, the findings also reveal substantial gaps in the current evidence base. The limited number of studies, small sample sizes, and methodological inconsistencies make it difficult to draw conclusions or establish standardized recommendations.

## Supplementary Information

Supplementary Material 1.

## Data Availability

No datasets were generated or analysed during the current study.

## Abbreviations

PA: Physical activity
6MWT: Six-minute walk test
CDT: Complete decongestive therapy
IPCT: Intermittent pneumatic compression therapy

## References

[1] Nicolás PC, Lipedema. More than a problem of fat legs. Update in the pathophysiology, diagnosis and surgical treatment. Revista de Cir. 2021;73(3):370–7.

[2] Buck DW, Herbst KL, Lipedema. A relatively common disease with extremely common misconceptions. Plast Reconstr Surg - Global Open. 2016;4(9). 10.1097/GOX.0000000000001043 PMC5055019 27757353

[3] Tuğral A, Bakar Y. An approach to lipedema: a literature review of current knowledge of an underestimated health problem. Eur J Plast Surg. 2019;42(6):549–58.

[4] Forner-Cordero I, Forner-Cordero A, Szolnoky G. Update in the management of lipedema. Int Angiol. 2021;40(4):345–57. 33870676 10.23736/S0392-9590.21.04604-6

[5] Wollina U, Heinig B. Differential diagnostics of lipedema and lymphedema: A practical guideline. Hautarzt. 2018;69(12):1039–47. 30402687 10.1007/s00105-018-4304-5

[6] Clarke C, Kirby JN, Smidt T, Best T. Stages of lipoedema: experiences of physical and mental health and health care. Qual Life Res. 2023;32(1):127–37. 35972618 10.1007/s11136-022-03216-w PMC9829602

[7] Aitzetmüller-Klietz ML, Busch L, Hamatschek M, Paul M, Schriek C, Wiebringhaus P et al. Understanding the vicious circle of Pain, physical Activity, and mental health in lipedema Patients—A response surface analysis. J Clin Med. 2023;12(16). 10.3390/jcm12165319 PMC10455654 37629361

[8] Hardy D, Williams A. Best practice guidelines for the management of lipoedema. Br J Community Nurs. 2017;22(Sup10):S44–8. 28961048 10.12968/bjcn.2017.22.Sup10.S44

[9] Organization WH. WHO guidelines on physical activity and sedentary behaviour. Geneva: World Health Organization; 2020. Available from: https://www.who.int/publications/i/item/9789240015128. 33369898

[10] Bull FC, Al-Ansari SS, Biddle S, Borodulin K, Buman MP, Cardon G, et al. World health organization 2020 guidelines on physical activity and sedentary behaviour. Br J Sports Med. 2020;54(24):1451–62. 33239350 10.1136/bjsports-2020-102955 PMC7719906

[11] Annunziata G, Paoli A, Manzi V, Camajani E, Laterza F, Verde L, et al. The role of physical exercise as a therapeutic tool to improve lipedema: A consensus statement from the Italian society of motor and sports sciences (Società Italiana Di scienze motorie e Sportive, SISMeS) and the Italian society of phlebology (Società Italiana Di Flebologia, SIF). Curr Obes Rep. 2024;13(4):667–79. 38958868 10.1007/s13679-024-00579-8 PMC11522091

[12] Erden E, Turk AC, Erden E, Yetim S, Borman P. The ıncidence of neuropathic pain and ıts ımpact on quality of life in patients with lipedema. Ir J Med Sci. 2025;194(5):1829–35. 40788448 10.1007/s11845-025-04024-0

[13] Prince SA, Cardilli L, Reed JL, Saunders TJ, Kite C, Douillette K, et al. A comparison of self-reported and device measured sedentary behaviour in adults: a systematic review and meta-analysis. Int J Behav Nutr Phys Act. 2020;17(1):31. 32131845 10.1186/s12966-020-00938-3 PMC7055033

[14] Hamilton DM, Haennel RG. Validity and reliability of the 6-minute walk test in a cardiac rehabilitation population. J Cardiopulm Rehabil. 2000;20(3):156–64. 10860197 10.1097/00008483-200005000-00003

[15] Peterson J, Pearce PF, Ferguson LA, Langford CA. Understanding scoping reviews: Definition, purpose, and process. J Am Assoc Nurse Pract. 2017;29(1):12–6. 27245885 10.1002/2327-6924.12380

[16] Tricco AC, Lillie E, Zarin W, O’Brien KK, Colquhoun H, Levac D, et al. PRISMA extension for scoping reviews (PRISMA-ScR): checklist and explanation. Ann Intern Med. 2018;169(7):467–73. 30178033 10.7326/M18-0850

[17] Forsberg C, Wengström Y. Att göra systematiska litteraturstudier: värdering, analys och presentation av omvårdnadsforskning. 3. uppl. ed. Stockholm: Natur & Kultur; 2013.

[18] Angst F, Lehmann S, Aeschlimann A, Sandòr PS, Wagner S. Cross-sectional validity and specificity of comprehensive measurement in lymphedema and lipedema of the lower extremity: a comparison of five outcome instruments. Health Qual Life Outcomes. 2020;18(1):245. 32698883 10.1186/s12955-020-01488-9 PMC7374881

[19] van Esch-Smeenge J, Damstra RJ, Hendrickx AA. Muscle strength and functional exercise capacity in patients with lipoedema and obesity: a comparative study. J Lymphoedema. 2017;12(1):27–31.

[20] Sakizli Erdal E, Ergin C, Haspolat M, Erturk B, Keser I. Effects of multimodal exercise program on edema, pain, exercise capacity, lower extremity muscle strength and function in patients with lipedema. Phlebology. 2025:2683555251343148. 10.1177/02683555251343148 40372970

[21] Atan T, Bahar-Ozdemir Y. The effects of complete decongestive therapy or intermittent pneumatic compression therapy or exercise only in the treatment of severe lipedema: A randomized controlled trial. Lymphatic Res Biology. 2021;19(1):86–95. 10.1089/lrb.2020.0019 33297826

[22] Benz T, Lehmann S, Sandor PS, Angst F. Relationship between subjectively-rated and objectively-tested physical function across six different medical diagnoses. J Rehabil Med. 2023;55:jrm9383. 38050460 10.2340/jrm.v55.9383 PMC10711800

[23] Mortimer PS, Pearson M, Gawrysiak P, Riches K, Keeley V, Tew KF, et al. LymphActiv: A digital physical activity behavior intervention for the treatment of lymphedema and lipedema. Lymphatic Res Biology. 2024;22(2):112–9. 10.1089/lrb.2023.0033 38394133

[24] Karas M, Bai J, Strączkiewicz M, Harezlak J, Glynn NW, Harris T, et al. Accelerometry data in health research: challenges and opportunities. Stat Biosci. 2019;11(2):210–37. 31762829 10.1007/s12561-018-9227-2 PMC6874221

[25] Du H, Newton PJ, Salamonson Y, Carrieri-Kohlman VL, Davidson PM. A review of the Six-Minute walk test: its implication as a Self-Administered assessment tool. Eur J Cardiovasc Nurs. 2009;8(1):2–8. 18694656 10.1016/j.ejcnurse.2008.07.001

[26] Cazzoletti L, Zanolin ME, Dorelli G, Ferrari P, Dalle Carbonare LG, Crisafulli E, et al. Six-minute walk distance in healthy subjects: reference standards from a general population sample. Respir Res. 2022;23(1):83. 35382813 10.1186/s12931-022-02003-y PMC8985335

[27] ATS statement. Guidelines for the six-minute walk test. Am J Respir Crit Care Med. 2002;166(1):111–7. 12091180 10.1164/ajrccm.166.1.at1102

