# Correction: objective measures of physical activity and physical capacities in lipedema - a scoping review

**DOI:** 10.1186/s12905-026-04345-x

**Published:** 2026-03-07

**Authors:** Ida Åström Malm, Paola Violasdotter Nilsson, Anita Hurtig-Wennlöf

**Affiliations:** 1 Department of Clinical Diagnostics, School of Health and Welfare, Jönköping University, Jönköping, Sweden; 2 Department of University Library, Jönköping University, Jönköping, Sweden

**Correction: Åström Malm et al. BMC Women's Health 26, 50 (2026)**

**https://doi.org/10.1186/s12905-026-04271-y**

In this article [1], the title was incorrectly given as ‘Objective measures of physical activity and physical capacities in lipedema - a scooping review’ but should have been ‘Objective measures of physical activity and physical capacities in lipedema - a scoping review’. 

Additionally, the family name and given name of the 2nd author Paola Violasdotter Nilsson has been corrected as below: 

Given name: Paola

Family Name: Violasdotter Nilsson
