# Supplementary material for: Objective measures of physical activity and physical capacities in lipedema - a scoping review
Source: BMC Womens Health. 2026 Jan 15;26:50. doi: 10.1186/s12905-026-04271-y (PMC12838409; doi:10.1186/s12905-026-04271-y)
Supplement: Supplementary file 1 — Supplementary Material 1. [file 12905_2026_4271_MOESM1_ESM.docx]

# **Lipedema and physical activity: search strategy** Databases searched

**AMED – The Allied and Complementary Medicine Database (EBSCOhost) n=3**Database coverage: 1995-
Date of last search: August 13, 2025
Search Mode: Advanced Search

|  | **Query** | **Results** |
| --- | --- | --- |
| #1 | TI ( lipedem* OR lipoedem* ) OR AB ( lipedem* OR lipoedem* ) OR SU ( lipedem* OR lipoedem* ) | 3 |

**CINAHL with FullText (EBSCOhost) n=197**Database coverage: 1981 -
Date of last search: August 13, 2025
Search Mode: Advanced Search/Proximity

|  | **Query** | **Results** |
| --- | --- | --- |
| #1 | (MH "Lipedema") | 83 |
| #2 | TI ( lipedem* OR lipoedem* ) OR AB ( lipedem* OR lipoedem* ) OR SU ( lipedem* OR lipoedem* ) | 197 |
| #3 | #1 OR #2 | 197 |

**MEDLINE (R) ALL (Ovid) n=648**Database coverage: 1946-
Date of last search: August 13, 2025
Search mode: Advanced search

|  | **Query** | **Results** |
| --- | --- | --- |
| #1 | Lipedema/ | 305 |
| #2 | (lipedem* or lipoedem*).tw,kf. | 631 |
| #3 | #1 or #2 | 648 |

**Scopus (Elsevier) n=1,077**Database coverage: 1788 -
Date of last search: August 13, 2025
Search Mode: Advanced search

|  | **Query** | **Results** |
| --- | --- | --- |
| #1 | TITLE-ABS-KEY ( lipedem* OR lipoedem* ) | 1,077 |

**Web of Science Core Collection* (Clarivate) n=730**Date of last search: August 13, 2025
Search Mode: Exact search

|  | **Query** | **Results** |
| --- | --- | --- |
| #1 | TS=(lipedem* OR lipoedem*) | 730 |

*Indexes included: Science Citation Index Expanded (SCI-EXPANDED)--1900-present, Social Sciences Citation Index (SSCI)--1900-present, Arts & Humanities Citation Index (AHCI)--1975-present, Conference Proceedings Citation Index – Science (CPCI-S)--1990-present, Conference Proceedings Citation Index – Social Science & Humanities (CPCI-SSH)--1990-present, Book Citation Index – Science (BKCI-S)--2005-present, Book Citation Index – Social Sciences & Humanities (BKCI-SSH)--2005-present, Emerging Sources Citation Index (ESCI)--2005-present, Current Chemical Reactions (CCR-EXPANDED)--1985-present, Index Chemicus (IC)--1993-present

## 
